# Discrete measurements of RNA polymerase and reverse transcriptase fidelity reveal evolutionary tuning

**DOI:** 10.1261/rna.080002.124

**Published:** 2024-09

**Authors:** Vladimir Potapov, Stanislas Krudup, Sean Maguire, Irem Unlu, Shengxi Guan, Jackson A. Buss, Benedict A. Smail, Trevor van Eeuwen, Martin S. Taylor, Kathleen H. Burns, Jennifer L. Ong, Robert J. Trachman

**Affiliations:** 1 New England Biolabs, Inc., Ipswich, Massachusetts 01938, USA; 2 École Supérieure de Biotechnologie de Strasbourg, 67400 Strasbourg, France; 3 Department of Pathology, Dana-Farber Cancer Institute, Boston, Massachusetts 02215, USA; 4 Laboratory of Cellular and Structural Biology, The Rockefeller University, New York, New York 10065, USA; 5 Department of Pathology, Mass General Brigham and Harvard Medical School, Boston, Massachusetts 02114, USA

**Keywords:** RNA polymerase, fidelity, reverse transcriptase, sequencing

## Abstract

Direct methods for determining the fidelity of DNA polymerases are robust, with relatively little sample manipulation before sequencing. In contrast, methods for measuring RNA polymerase and reverse transcriptase fidelities are complicated by additional preparation steps that introduce ambiguity and error. Here, we describe a sequencing method, termed Roll-Seq, for simultaneously determining the individual fidelities of RNA polymerases and reverse transcriptases (RT) using Pacific Biosciences single molecule real-time sequencing. By using reverse transcriptases with high rolling-circle activity, Roll-Seq generates long concatemeric cDNA from a circular RNA template. To discern the origin of a mutation, errors are recorded and determined to occur within a single concatemer (reverse transcriptase error) or all concatemers (RNA polymerase error) over the cDNA strand. We used Roll-Seq to measure the fidelities of T7 RNA polymerases, a Group II intron-encoded RT (Induro), and two LINE RTs (*Fasciolopsis buski* R2-RT and human LINE-1). Substitution rates for Induro and R2-RT are the same for cDNA and second-strand synthesis while LINE-1 has 2.5-fold lower fidelity when performing second-strand synthesis. Deletion and insertion rates increase for all RTs during second-strand synthesis. In addition, we find that a structured RNA template impacts fidelity for both RNA polymerase and RT. The accuracy and precision of Roll-Seq enable this method to be applied as a complementary analysis to structural and mechanistic characterization of RNA polymerases and reverse transcriptases or as a screening method for RNAP and RT fidelity.

## INTRODUCTION

Nucleic acid polymerases are tuned to transfer information with fidelity while generating mutations to promote diversity. Each polymerase evolved to uniquely balance these contradictory traits. High-fidelity DNA polymerases have captured attention owing to their genomic impact and mutagenic signatures associated with cancer, aging, and exposures. However, lower-fidelity polymerases play an important biological and biotechnological role. RNA polymerase infidelity enables diversity of phenotype expression within an organism (Gordon et al. 2015; Romero Romero et al. 2022) and is an important consideration in therapeutic mRNA production. Reverse transcriptases (RTs) are essential for retroviral and transposon replication, thus requiring precise tuning of error rates. Despite the vast biological importance of RNA polymerases and reverse transcriptases, techniques for assessing their fidelities are limited and lacking.

**FIGURE 1.**
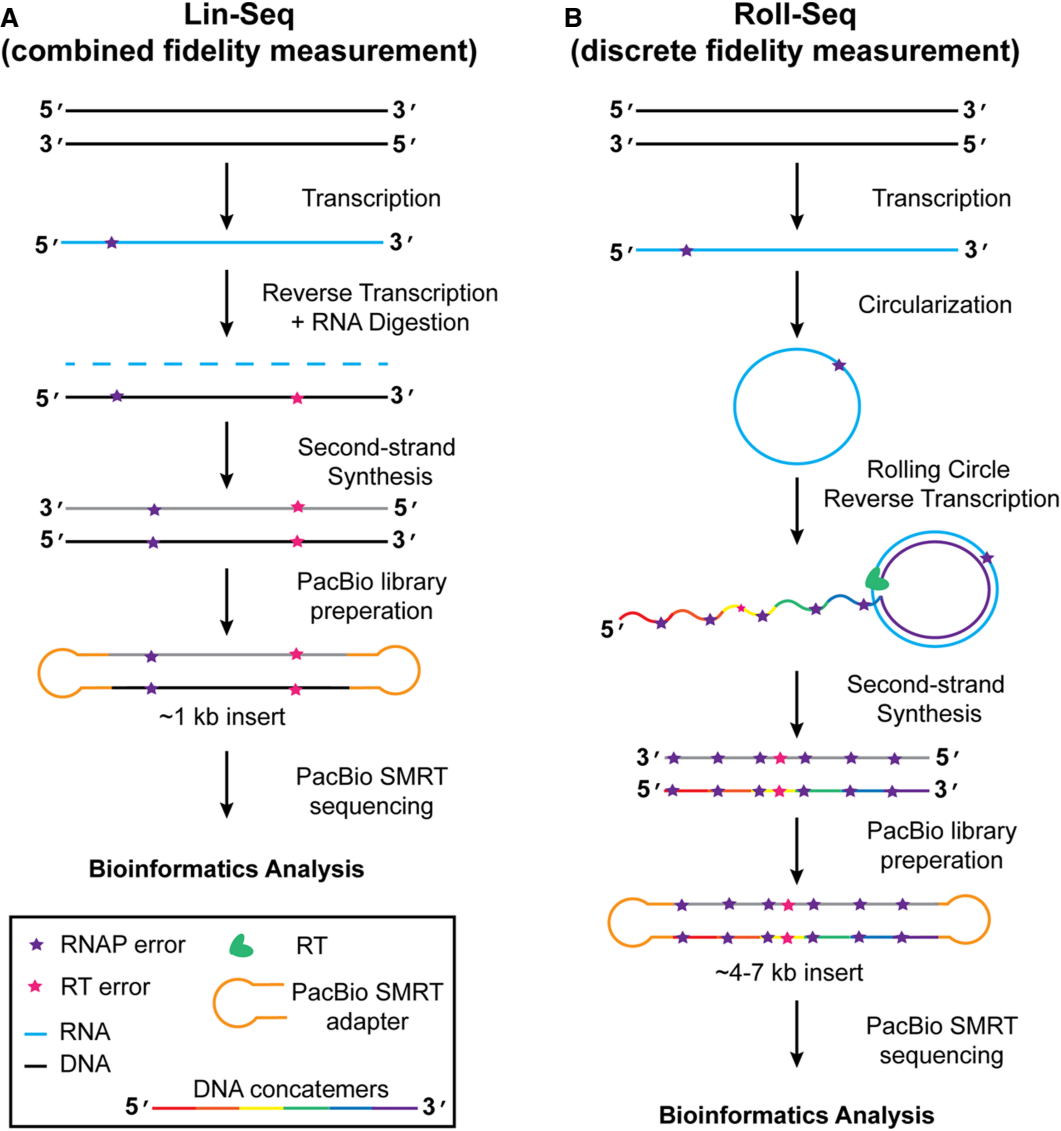
Comparison of linear sequencing and Roll-Seq workflows. (*A*) Linear sequencing protocol workflow showing the generation of RNA from DNA, and subsequent generation of cDNA followed by second-strand synthesis for input into PacBio preparation. Polymerase errors that arise during transcription (purple star) cannot be distinguished from errors arising during reverse transcription (pink star) during bioinformatic analysis. (*B*) Roll-Seq workflow. A DNA template, containing coding regions for a Group I ribozyme on 5′ and 3′ ends, is transcribed by an RNA polymerase that will make random errors (purple star). Ribozyme reaction results in the circularization of the RNA. Rolling-circle reverse transcription results in multiple concatemers (rainbow cDNA). Errors originating from transcription (purple star) appear at the same location in every concatemer. Errors from reverse transcription are randomly distributed throughout the cDNA.

Several methods have attempted to measure RNA polymerase and RT fidelities, each with varying degrees of success. Before next-generation sequencing, nucleotide misincorporation assays were the standard for measuring polymerase fidelities (Huang and Sousa 2000; Skasko et al. 2005; Jamburuthugoda and Eickbush 2011). However, these methods can only detect misincorporation with a limited set of nucleotides present, which is an artificial set of conditions. The advent of next-generation sequencing provided a tool for direct measurement of polymerase fidelity. However, challenges persist. While error rates of DNA polymerases are readily determined, the low accuracy of direct RNA sequencing complicates RNA polymerase fidelity measurements. The previously developed linear sequencing protocol generates cDNA from RNA polymerase product using an RT (Potapov et al. 2018; Chen et al. 2022). Methods such as these result in a combined RNA polymerase and RT error rate measurement (Fig. 1A; Yasukawa et al. 2017). A major advancement resulted when Gout and colleagues used barcoded RNA tags followed by reverse transcription and Illumina sequencing to distinguish RNA polymerase errors from errors generated by RT and library preparation (Gout et al. 2013). Subsequent studies were able to measure RNA polymerase fidelities (Gout et al. 2017; Reid-Bayliss and Loeb 2017), or low copy viral mutations (CirSeq) (Whitfield and Andino 2016), by performing rolling-circle reverse transcription from circular RNA templates (Gout et al. 2017s; Reid-Bayliss and Loeb 2017). Due to PCR amplification after cDNA synthesis, RT fidelities were intractable, and the measurement accuracy was compromised.

In the current study, we develop a method to differentiate errors deriving from RNA polymerase or RT using Pacific Biosciences single molecule real-time (SMRT) sequencing platform (Fig. 1B). Previous methods performing rolling-circle consensus sequencing implemented M-MuLV-based RTs that are suboptimal at rolling-circle reverse transcription. To improve upon this technique, RTs with elevated rolling-circle activity were chosen to generate long concatemeric cDNA. As a result, cDNA products were prepared without the need for amplification, allowing for direct measurement of RNA polymerase and reverse transcriptase error rates. Error rates of a Group II intron-encoded RT (Induro), and the non-LTR RTs from human LINE-1 (L1-RT, also referred to as ORF2p) and a novel R2-RT (FBu) were compared. Among these three enzymes, we observed a broad range of error rates with distinct error profiles. cDNA and second-strand synthesis error rates were compared for the RT series revealing that L1-RT has >2.5-fold increase in error rate on a DNA template. We also performed Roll-Seq on a translatable circular RNA template revealing that structured RNA reduces both transcription and reverse transcription fidelity. Thus, discrete fidelity measurements by Roll-Seq complement structural and mechanistic information performed on RTs.

## RESULTS

### Identification of reverse transcriptases with rolling-circle activity

The Roll-Seq workflow requires reverse transcriptases (RTs) to perform rolling-circle reverse transcription—a process where RTs make multiple passes along a circular RNA template while strand displacing cDNA. The ability of an RT to perform rolling-circle reverse transcription can generally be rationalized by the biological activities it performs. Homologs of viral M-MuLV RTs are not proficient at rolling-circle reverse transcription and are especially challenged by long circular RNA. Retroviral RTs are not required to strand displace in vivo and are not required to reverse transcribe exceptionally complex templates. Group II intron RTs are adept at rolling-circle reverse transcription (Lambowitz and Zimmerly 2011; Maguire and Guan 2022), a characteristic likely deriving from the necessity to reverse transcribe through highly structured RNA templates (Zhao and Pyle 2017). We hypothesized that long interspersed nuclear element (LINE) RTs would readily perform rolling-circle reverse transcription given that LINE propagation requires reverse transcription through multiple highly structured RNA elements (Christensen et al. 2006). Furthermore, these RTs are required to perform second-strand DNA synthesis while strand displacing RNA (Kurzynska-Kokorniak et al. 2007; Han 2010).

To identify reverse transcriptases that perform rolling-circle reverse transcription, we tested the activity of reverse transcriptases on both a linear and a circular RNA template of ∼1 kb (Supplemental Fig. S1). The LINE RTs, R2-RT from *Bombyx mori* (BMo) and human LINE-1open reading frame 2 protein (ORF2p) RT (L1-RT) (Sakaki et al. 1986; Burke et al. 1987), perform varying degrees of rolling-circle reverse transcription relative to our negative and positive controls, ProtoScript II RT and Induro RT, respectively. While the yield and length of cDNA from L1-RT reactions appeared sufficient, BMo rolling-circle activity was insufficient for Roll-Seq analysis.

To increase the scope of comparison between RTs and further understand the difference in fidelity between model mobile genetic elements, we sought to identify an R2-RT that is highly active in rolling-circle reverse transcription. Screening of uncharacterized R2-RT homologs with sequence similarity of <80% to BMo, identified a highly active R2-RT from the intestinal fluke *Fasciolopsis buski* (FBu) (Supplemental Fig. S2). FBu produces higher cDNA yields at lower temperatures (36°C–40°C) than all RTs tested (Supplemental Fig. S1B,C). While maintaining many of the characteristics of BMo, FBu generates longer cDNA by rolling-circle reverse transcription, in higher yield. These results demonstrate that FBu is sufficiently active to perform Roll-Seq.

### Discrete fidelity measurements using Roll-Seq

The Roll-Seq method was designed to measure the fidelity of both RNA polymerases and RTs with precision and accuracy. We hypothesized that the high accuracy of Pacific Biosciences long-read sequencing in conjunction with highly concatemerized cDNA generated by rolling-circle reverse transcription would enable us to differentiate errors generated by RNA polymerases and reverse transcriptases. Roll-Seq requires a minimum of three passes around a circular RNA template and should not exceed 10 passes for an ∼1 kb template. The upper limit of concatemers is dictated by the length of the template, which determines the number of passes the sequencing polymerase can make to generate consensus. By limiting the analysis between 3 and 10 concatemers, 80%–95% of sequencing reads have an accuracy of Q40 or greater (Supplemental Fig. S3). To discern the origin of a mutation, errors are recorded and determined to occur within a single concatemer (reverse transcriptase error) or all concatemers (RNA polymerase error) over the cDNA strand. These data enable direct measurement of substitution, deletion, and insertion errors with high accuracy.

**FIGURE 2.**
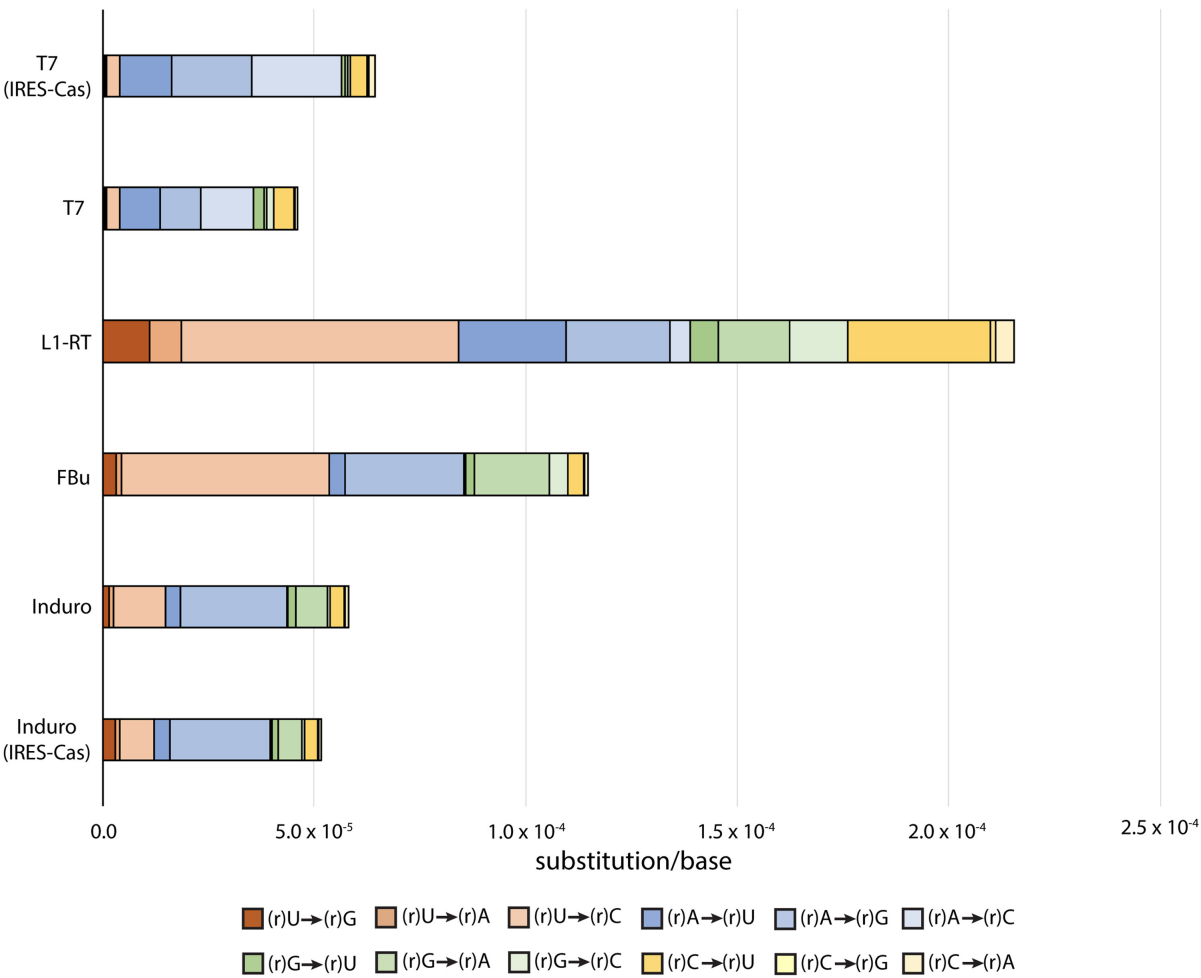
Substitution error spectrum for RNA polymerase and cDNA synthesis by RT enzymes. Unless noted, the template sequence used for fidelity measurement is DNA-1 (Supplemental Table S1). Substitutions key shows RNA polymerase errors. Errors for reverse transcriptases occur for the deoxyribose form of the base with uracil being replaced by thymine.

To test the ability of Roll-Seq to discern RNA polymerase errors from RT errors, and to compare error profiles between these groups, we performed Roll-Seq with T7 RNA polymerase (T7) and three reverse transcriptases (Induro, L1-RT, and FBu). The error rates of T7 did not change when coupled to different reverse transcriptases (Supplemental Table S2). While the rates of insertion and deletion are similar for all of the polymerases, the rates of substitution, and the substitution profiles, vary significantly (Fig. 2). Overall, the substitution profiles determined by Roll-Seq are consistent with previous measurements using a combined fidelity assay using T7 RNA polymerase and the M-MuLV variant, ProtoScript II (Potapov et al. 2018).

To confirm that the individual fidelities of Roll-Seq are additive and consistent with other fidelity measurement methods, we performed fidelity measurements as described in Potapov et al. (2018) for comparison, and now refer to this technique as the linear sequencing protocol in the current manuscript. As expected, the difference in overall fidelity between the linear sequencing protocol and the sum of RNA polymerase and RT fidelities of Roll-Seq is modest and within error of the measurements (Supplemental Fig. S4).

### Fidelity of T7 RNA polymerase

**FIGURE 3.**
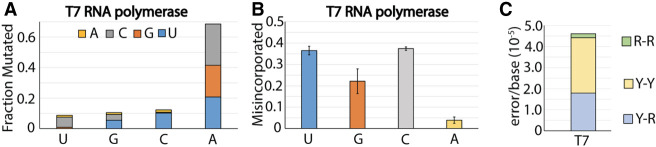
Breakdown of substitution mutations of T7 and T7^Y639F^ RNA polymerase. (*A*) Fraction of substitution error generated for each nucleotide by T7 RNA polymerase. Fractions are subdivided by the proportion of each misincorporated nucleotide adenine (yellow), cytosine (gray), guanine (orange), and uridine (blue). (*B*) Fraction of misincorporation for each nucleotide by T7 RNA polymerase. (*C*) Error rate for T7 RNA polymerase subdivided by transversion (yellow and green) and transition (blue) mutations.

T7 RNA polymerase performs most industrial RNA synthesis for therapeutic mRNA. To understand the context of errors introduced by T7 RNA polymerase we performed Roll-Seq using T7. Substitutions occur at a rate of 3.8 × 10^−5^ error/base, accounting for 71% of the total T7 mutations (Table 1). Surprisingly, 68% of substitution errors occur in place of adenine (Fig. 3A). Substitution of adenine is equally probable between cytosine, guanine, and uridine. Substitution of cytosine, guanine, and uridine range from 7.5% to 11%. Misincorporated bases are overwhelmingly pyrimidine nucleotides, uridine (37%) and cytosine (37%), with adenine accounting for only 4% of misincorporation (Fig. 3B). Together, these data reveal that T7 is proficient at polymerizing pyrimidine bases and deficient at incorporating adenine.

**TABLE 1.** Error rates (×10^−6^) for polymerases

Enzyme	Substitution	Deletion	Insertion	Total
T7	38 ± 5	2 ± 0	13 ± 1	53 ± 6
Induro	57 ± 3	6 ± 1	49 ± 4	113 ± 2
L1-RT	220 ± 4	9 ± 0	49 ± 2	278 ± 6
FBu	117 ± 67	7 ± 1	52 ± 12	175 ± 62

### Fidelity of reverse transcriptases

To understand the range of fidelities among a set of evolutionarily divergent RTs, we performed Roll-Seq using the RTs Induro, L1-RT, and FBu. An error is assigned to the RT if a substitution, insertion, or deletion is reported in a single concatemer of a cDNA containing ≥3 concatemers. This condition ensures that errors are arising from the RT and not from the RNA polymerase or the sequencing polymerase.

**FIGURE 4.**
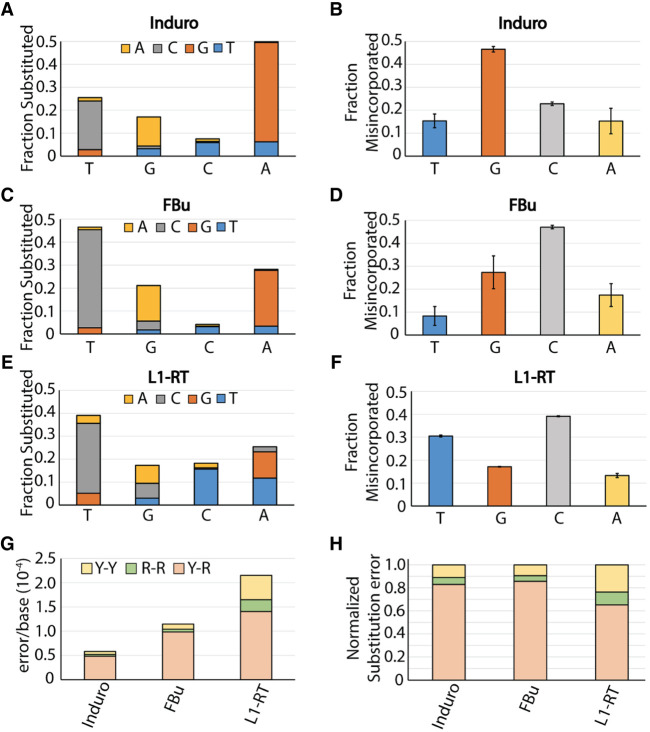
Breakdown of substitution mutations for reverse transcriptases. (*A*) Fraction of substitution error generated for each nucleotide by Induro. Fractions are subdivided by the proportion of misincorporation for adenine (yellow), cytosine (gray), guanine (orange), and thymine (blue). (*B*) Fraction of misincorporation for each nucleotide by Induro. (*C*) Fraction of substitution error generated at each cognate nucleotide by FBu. Fractions are subdivided by the proportion of misincorporation for adenine (yellow), cytosine (gray), guanine (orange), and thymine (blue). (*D*) Fraction of misincorporation for each nucleotide by FBu. (*E*) Fraction of substitution error generated at each cognate nucleotide by L1-RT. Fractions are subdivided by the proportion of misincorporation for adenine (yellow), cytosine (gray), guanine (orange), and thymine (blue). (*F*) Fraction of misincorporation for each nucleotide by L1-RT. (*G*) Substitution error rates of RTs subdivided by mutation type. (*H*) Normalized substitution errors by type.

RT substitution errors range from 5.7 × 10^−5^ errors/base to 2.2 × 10^−4^ errors/base for Induro and L1-RT, respectively: a fourfold difference in substitution rate (Table 1). Substitution of adenine accounts for 49% of Induro substitution mutations while thymine is substituted at a rate of 40% and 47% for L1-RT and FBu (Fig. 4A–F). The most commonly misincorporated base for Induro is guanine (Fig. 4B) while L1-RT and FBu misincorporate cytosine at the greatest rate (Fig. 4D,F). Overall, the substitution profiles of L1-RT and FBu share greater resemblance to each other than Induro. However, there are differences among all three RTs. Substitution of cytosine is fourfold higher for L1-RT than it is to FBu, resulting in a threefold higher fraction of misincorporation of thymine. Additionally, the types of mutations made between RTs vary. Transitions are the predominant mutation type for all three RTs (Fig. 4G). The fraction of transversion mutations for L1-RT is double that of Induro and FBu (Fig. 4H). The error rate resulting in a purine–purine mismatch for L1-RT is 2.5 × 10^−5^. This is nearly half the total substitution rate of Induro RT. The high overall error rate of L1-RT combined with its tendency to generate purine–purine (R–R) mismatches suggests a more open active site for this enzyme.

### Measurement of insertion and deletion error rates

Both sequencing by synthesis methods, and nanopore technologies suffer from low accuracy within homopolymer sequences. Homopolymer sequences cause slippage of polymerases (Liu and Martin 2009) resulting in a higher frequency of insertions and deletions. Pacific Biosciences SMRT sequencing reduces the error associated with homopolymer sites by generating a consensus sequence resulting from multiple passes along the sample template sequence. By generating multiple concatemers within a cDNA it is possible to determine if an insertion, or deletion, is made in every base combination.

**FIGURE 5.**
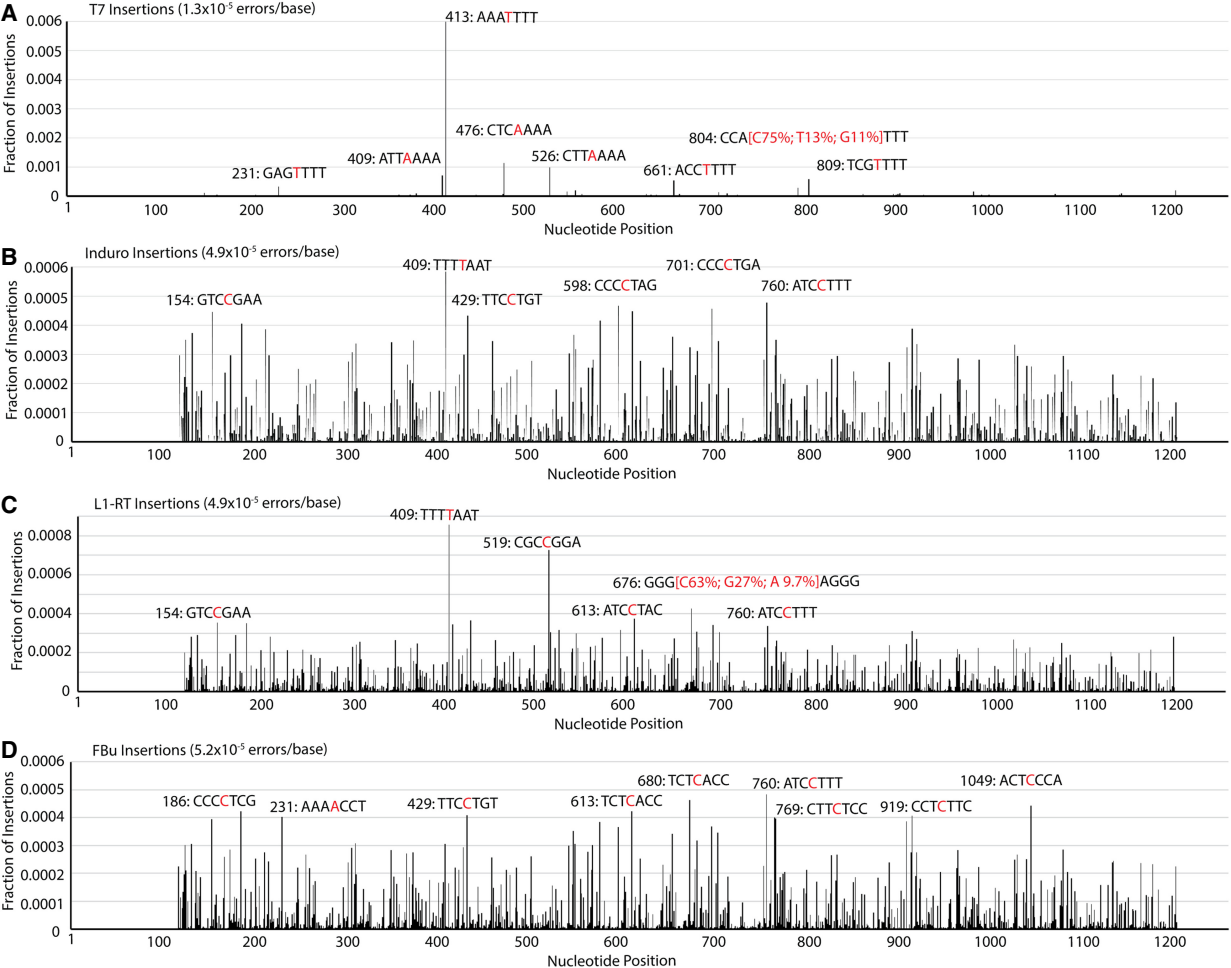
Fraction of insertion mutations at each nucleotide position on template DNA1 for (*A*) T7, (*B*) Induro, (*C*) L1-RT, and (*D*) FBu. Most prevalent insertions are labeled red with three adjacent nucleotides shown in black.

The rate of insertion for T7 (1.3 × 10^−5^ errors/base) is sixfold greater than the rate of deletion (Table 1; Fig. 5A; Supplemental Fig. S5A). Insertions are five times more prevalent for RTs than T7, while deletions are two to three times more prevalent in RTs than T7 (Table 1). While the insertion distribution of the RNA polymerase is biased toward a few locations, the insertions observed for the RTs are distributed over the sequence (Fig. 5C,D). Mapping the six most prevalent insertions for each RT onto the DNA1 sequence shows that cytosine is the most inserted base, followed by thymine and adenine. RT deletions are clustered and occur at the same location in the DNA1 sequence (Supplemental Fig. S5). The deletion sites on the DNA1 template occur at homopolymer tracts for both the RTs and T7, conferring that polymerases are generally challenged by slippery sequences.

### Comparison of reverse transcriptase cDNA and second-strand synthesis

**FIGURE 6.**
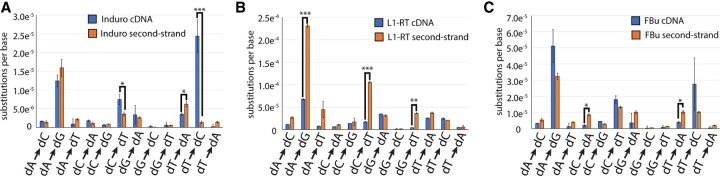
Comparison of substitutions for cDNA and second-strand synthesis for (*A*) Induro, (*B*) L1-RT, and (*C*) FBu. Error bars report standard error, while asterisks indicate two-way ANOVA.

Many retrotransposon and retroviral RTs produce dsDNA from a cDNA template in a process termed second-strand synthesis (Cost et al. 2002; Basu et al. 2008; Khadgi et al. 2019). To compare the fidelity of RTs during the second phase of a retrotransposition cycle, we modified the analysis from the linear sequencing protocol method to directly examine differences between top (cDNA) and bottom strands (second strand) and compared these values to those obtained by Roll-Seq (Fig. 6; Table 2). Group II intron-encoded RTs do not perform second-strand synthesis during self-replication, relying on host machinery to complete replication (Smith et al. 2005). However, Induro has the highest second-strand synthesis fidelity among the three RTs tested. The substitution error rate of second-strand synthesis is nearly identical to that of cDNA synthesis for both Induro (5.7 × 10^−5^ errors/base) and FBu (1.17 × 10^−4^ errors/base). This contrasts with L1-RT which has a 2.5-fold increase in substitution rate from cDNA to second-strand DNA synthesis. Much like substitution errors, L1-RT is the only RT with a significant change in insertion rate (4.9 × 10^−5^ to 1.32 × 10^−4^ error/base). Deletions are the least prevalent mutation for all three RTs in both cDNA and second-strand synthesis, ranging from 6 × 10^−6^ to 2.8 × 10^−5^ errors/base. However, the deletion rate increases for all three RTs compared to cDNA synthesis. These data indicate that a DNA template causes more slippage relative to an RNA template.

**TABLE 2.** Error rates (×10^−6^) for cDNA and second-strand synthesis

Enzyme	Synthesis	Substitution	Deletion	Insertion	Total
Induro	cDNA	57 ± 3	6 ± 1	49 ± 4	113 ± 2
	Second strand	37 ± 5	28 ± 4	52 ± 2	117 ± 10
L1-RT	cDNA	220 ± 4	9 ± 0	49 ± 2	278 ± 6
	Second strand	567 ± 40	15 ± 4	132 ± 5	714 ± 47
FBu	cDNA	117 ± 67	7 ± 1	52 ± 12	175 ± 62
	Second strand	101 ± 15	18 ± 3	73 ± 8	192 ± 22

### Measurement of error rate on a translatable template

**FIGURE 7.**
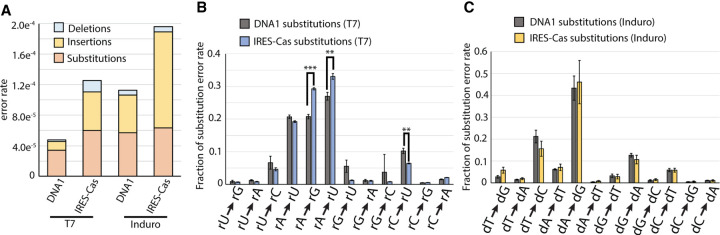
Comparison of substitution rates on template DNA1. (*A*) Error rates for T7 and Induro on DNA1 and IRES-Cas subdivided by error type. Substitution error profiles of DNA1 and IRES-Cas for (*A*) T7 RNA polymerase and (*B*) Induro RT. (*C*) Fraction of substitution errors for Induro using the DNA1 (gray) or IRES-Cas (yellow) template.

To test the consistency of polymerase fidelities on different sequences, we performed Roll-Seq with T7 RNAP and Induro RT on a functional template containing structured RNA and an open reading frame. The total error rate of both transcription and reverse transcription increases for a template containing a cardiovirus type II IRES and Cas9 open reading frame (IRES-Cas) (Fig. 7A). The fidelity of T7 is reduced by >50% on the IRES-Cas template with impact on substitution (3.8 × 10^−5^ errors/base → 6.0 × 10^−5^ errors/base), deletion (2.1 × 10^−6^ errors/base → 1.5 × 10^−5^ errors/base), and insertion (1.3 × 10^−5^ errors/base → 5.1 × 10^−5^errors/base) error rates. The total error rate of Induro RT increases from 1.1 × 10^−4^ → 2.0 × 10^−4^ errors/base, with nearly the entire reduction in fidelity being accounted by an increased rate of insertion (4.9 × 10^−5^ errors/base → 1.2 × 10^−4^ errors/base) (Supplemental Table S2). The cause of the increased substitution rate of T7 RNAP is not apparent. This increased rate of substitution is only observed for the transition rA → rG and transversion rA → rC (Fig. 7B). These results cannot be attributed to an imbalance of the nucleotide pool, given that adenine is reduced in frequency by 2% relative to the ideal 25%, the rA → rU substitution rate is unchanged, and the adenine substitution rate does not change for Induro RT (Fig. 6C). Additionally, the frequency of substitution is evenly distributed over the entire IRES-Cas sequence (Supplemental Fig. S6), suggesting that errors are not present in the initial template or localized to a single region of the template. Together these findings rule out nucleotide pool bias and errors within the starting DNA template from accounting for the observed differences in fidelity on the two sequences analyzed in this study.

## DISCUSSION

In this study, we performed Pacific Biosciences SMRT sequencing on cDNA generated by rolling-circle reverse transcription to distinguish the individual error rates of RNA polymerase and RTs. The origin of polymerase error was determined to result from the RNA polymerase if the mutation is observed in every instance of cDNA concatemer, or from the RT if the mutation occurs in a single instance within the cDNA. A broad range of fidelities were observed over the polymerases surveyed. High-accuracy individual fidelity measurements of RNA polymerase and RTs complement structural and mechanistic studies of polymerases, providing new insight into the tuning of fidelity for biological activity.

T7 RNAP polymerase is the most popular RNA polymerase for performing enzymatic RNA synthesis in vitro. We found that transcriptions performed with T7 RNAP (HiScribe kit) result in total error rates of 5.3 × 10^−5^ errors/base for the DNA1 template, with 68% of substitution mutations occurring for adenine. A recent report using a new circular RNA sequencing method, tARC-Seq, reported that the T7-produced Pfizer SARS-CoV-2 mRNA vaccine contains 2.3 × 10^−4^ error/base, with rG → rA being the most prevalent substitution (Bradley et al. 2022). The discrepancy in these results likely arises from vastly different transcription conditions, unbalanced nucleotide composition of the SARS-CoV-2 mRNA template, and lower accuracy of Illumina sequencing. To this point, the error rate of T7 RNAP on the IRES-Cas template was 1.26 × 10^−4^ errors/base, suggesting that the template sequence might be the most dominant contributing factor to RNA polymerase fidelity. The ability to determine the fidelity bias of T7 RNAP with high accuracy provides an opportunity to improve template design and transcription conditions for mRNA therapeutics as well as to screen for high-fidelity T7 RNAP variants.

The main advancement of Roll-Seq over other rolling-circle-based sequencing methods is the application of RTs that generate long concatemeric cDNA coupled to Pacific Biosciences SMRT sequencing. Group II intron-encoded RTs have previously been shown to function well in rolling-circle reverse transcription. This trait is attributed to their requirement to reverse transcribe through folded, structured RNA. We correctly postulated that LINE RTs would also be proficient at rolling-circle reverse transcription given their biological function may necessitate strand displacing thousands of DNA/RNA hybrid base pairs (Eickbush and Eickbush 2015), albeit with support from RNase H2 in the case of L1-RT (Benitez-Guijarro et al. 2018). Establishing these activities allowed us to compare the fidelities between a Group II intron-encoded RT and LINE RTs.

The ability to determine the fidelity of reverse transcriptases with high accuracy is unique to this work. Only inferred error rates of RTs have been reported for short tandem repeats (Fungtammasan et al. 2016), in addition to combined RNA polymerase/RT error rates. Roll-Seq enabled comparison between cDNA synthesis fidelities in the absence of RNA polymerase or amplification background error. The substitution error rate of cDNA synthesis for the RT series is broad, ranging from 5.7 × 10^−5^ to 2.2 × 10^−4^ errors/base. These values were determined at dNTP concentrations consistent with cancerous cells (Traut 1994). It is reasonable to consider that RT error rates may be lower in senescent cells that possess dNTP concentrations two orders of magnitude below those found in cancerous cells.

The difference in cDNA and second-strand synthesis fidelities revealed a disparity between RTs. Induro and FBu have only a modest difference in error rate between cDNA and second-strand synthesis. However, L1-RT has a significant increase in substitutions and indels when performing second-strand synthesis. The increased slippage observed for L1-RT along with the higher propensity to form purine–purine mismatches is consistent with an open active site with poorly coordinated substrates. While crystal structures of HIV-1 RT with active-site RNA/DNA and DNA/DNA hybrids reveal only subtle structural differences between these two states (Xavier Ruiz and Arnold 2020), solution studies of this enzyme reveal a number of dynamic orientational conformations during synthesis, including flipping and sliding, during synthesis. Viral RTs and human non-LTR retrotransposon RTs also have significant structural deviations from L1 other LTR RTs and group II introns including the absence of an N-terminal extension (NTE) that allows distinct enzymatic activities like template jumping (Baldwin et al. 2024), yet there is a lack of structural characterization of these latter RTs in second-strand synthesis. R2-RTs are incorporated at specific genomic locations and are faithfully copied over multiple replications (Wilkinson et al. 2023; Baldwin et al. 2024), L1-RT, which jumps throughout the genome, possesses unique fidelity properties that may expand genetic diversity (Baldwin et al. 2024).

By changing the template sequence from the idealized DNA1 to the functional IRES-Cas sequence encoding structured RNA and an open reading frame, there was reduced fidelity for both RNA polymerase and RTs. The rate of substitution increased for T7 RNAP on the functional IRES-Cas template, but not for Induro RT. Similar results were observed with the tARC-Seq method, where T7 error rates increased on the wild-type S gene of SARS-CoV-2 relative to the codon-optimized mRNA vaccine (Bradley et al. 2022). Natural RNA sequences contain functional, structured RNA segments. Both RNA polymerase and RT are impacted by these structured regions. In the case of RNA polymerases, riboswitches or hairpins can cotranscriptionally fold to influence the fate of downstream RNA polymerase activities (Hartvig and Christiansen 1996; Jones and Ferré-D'Amaré 2015). These motifs often reside upstream of poly(U) sequences as expression platforms or termination sequences, respectively (Roth and Breaker 2009; Calvopina-Chavez et al. 2022). Poly(U) sequences are known to destabilize RNA/DNA hybrids and cause the polymerase to switch between elongation and initiation (Lyakhov et al. 1998). Here, we observed that these sequences are also mutational hot spots, providing a mutational pathway to bypass pausing. While less studied, structured RNAs also alter the rate of reverse transcription (Shaw et al. 2023). Our results demonstrated that structured RNA increases the frequency of mutation for both T7 RNAP and Induro RT, particularly for insertions at homopolymer sequences.

In conclusion, we developed a method to simultaneously determine the fidelities of RNA polymerases and RTs. This method is capable of streamlining the screening of RNAP and, RT polymerases for optimal fidelity. The data from this method also provide a fidelity-based perspective on the product of RNA polymerases and RTs to complement structural and mechanistic studies. Future work will seek to expand upon this RT series, and other studies (Penno et al. 2017), by varying dNTP concentration to mimic different cell states, and further challenge polymerases by more complex structured RNA templates.

## MATERIALS AND METHODS

Unless otherwise stated, all reagents are from New England Biolabs. All oligonucleotides were synthesized by Integrated DNA Technologies.

### Reverse transcriptase expression and purification

FBu and human LINE-1 RT were cloned, expressed, and purified for this study. L1-RT was expressed and purified as described previously (Baldwin et al. 2024). Briefly, LINE-1 ORF2p “core” (residues 238–1061, comprising tower, RT, and wrist domains but lacking EN and CTD) was expressed and purified from *Escherichia coli* as an N-terminal His6-MBP-3C fusion, lysed using a microfluidizer, purified by sequential Ni-NTA and heparin affinity tag cleaved using 3C protease, tag removed using heparin affinity, and polished on gel filtration using a Superdex 200 column (Cytiva) in a final buffer containing 500 mM NaCl, 5% glycerol, 2 mM MgCl_2_, 0.5 mM TCEP, and 20 mM HEPES pH 8.0.

FBu was cloned into pMal-c5e. Plasmid was transformed into *E. coli* C3013 cells (NEB) and grown on LB-Lennox agar plates supplemented with 50 µg/mL ampicillin overnight. Three to four colonies were used to inoculate 8 mL of LB-Lennox supplemented with 50 µg/mL ampicillin, and the culture was shaken at 30°C for 7 h. One milliliter of starter culture was used to inoculate 1 L LB-Amp. The culture was incubated at 20°C for 12 h before induction with 0.3 mM IPTG and incubation at 16°C for 8 h. Cells were harvested by centrifuge at 4k RPM at 4°C. Cell pellets were resuspended in Lysis Buffer (50 mM Tris pH 7.5, 1 M NaCl, 2 mM MgCl_2_, 5 mM DTT, 10% glycerol) in 1× protease inhibitor cocktail (Roche). Cells were lysed by sonication for 1 min on ice. Lysate was clarified by centrifugation and passed over a 5 mL MBPTrap (Cytiva) column. FBu was eluted in Lysis Buffer supplemented with 30 mM Maltose. Eluent was then diluted fivefold with DEPC-treated H_2_O and purified by heparin affinity column. Fractions containing purified protein were pooled, concentrated, and exchanged into a buffer containing 50 mM Tris-HCl pH 7.5, 500 mM NaCl, 1 mM MgCl_2_, 5 mM DTT before being diluted twofold with 100% glycerol.

### In vitro transcription

Designed sequence DNA1 (Supplemental Table S1) was cloned into the pUC18 vector. Plasmid was linearized by mixing 20 µg plasmid, 100 U HpaI (DNA-1) or 100 U XbaI (IRES-Cas), and 1× Thermo Pol. Buffer at a final volume of 1 mL, followed by incubation at 37°C for 1 h. The restriction digest reaction was then supplemented with 20 μL PreCR Repair Mix, 0.1 mM each dNTP, 0.5 mM NAD^+^, and 1× Thermo Pol. Buffer. The solution was incubated at 37°C for 30 min. Templates were purified using the Monarch PCR and DNA Cleanup Kit. Five micrograms of linearized, PreCR-treated plasmid was used to transcribe RNA using the HiScribe T7 High Yield RNA Synthesis kit (NEB). After transcription, 1× DNase I buffer and 4 U DNase I were added, followed by incubation at 37°C for 20 min. Samples were purified using the Monarch RNA Cleanup kit (NEB). Sample concentration and quality were determined using a NanoDrop 1000, Qubit 4 fluorometer, and Agilent TapeStation.

### Reverse transcription assays

cDNA synthesis for all commercial enzymes was carried out using the manufacturer's specifications except for when noted for temperature gradients. Ten micrograms of RNA template and 0.5 µM reverse transcriptase primer (Supplemental Table S1) were mixed and annealed at 65°C for 5 min followed by cooling on bench for 5 min. 1× Induro Buffer supplemented with 3 mM MgCl_2_ and 1 U of Induro RT or 125 ng of LINE-1/FBu RT enzyme were mixed with circular RNA and primer mix and incubated at 37°C for 5 min followed by incubation at 4°C for 10 min. One millimolar from each dNTP was added to initiate the reaction. Reactions were incubated for 1 h or for 3–5 min for linear sequencing protocol and Roll-Seq, respectively.

#### Combined fidelity sequencing preparation

The linear sequencing protocol was performed as described by Potapov et al. (2018). Briefly, cDNA synthesis was performed using 10 μg of linear RNA template followed by digestion with RNase H. After purification using the Monarch PCR and DNA Cleanup Kit, second-strand synthesis was performed using ProtoScript II reverse transcriptase. The reaction was purified using the Monarch PCR and DNA Cleanup Kit (NEB). Double-stranded DNA was prepared for sequencing using the SMRTbell prep kit 3.0 (Pacific Biosciences) and SMRTbell barcoded adapter plate 3.0 (Pacific Biosciences). After barcode addition, Sequel II binding kit 3.1 (Pacific Biosciences) was used to anneal primer and polymerase. Libraries were run on a Pacific Biosciences Sequel II instrument collecting HiFi reads with 2 h pre-extension time and 30 h movie length.

#### Roll-Seq protocol

Roll-Seq protocol was performed in five phases including the (i) synthesis of cDNA originating from a circular RNA template, (ii) second-strand synthesis of the complementary cDNA strand, (iii) addition of SMRTbell barcoded adapters to the dsDNA sample, (iv) size selection by Sage Science Blue Pippin, and (v) long-read sequencing using a Pacific Biosciences Sequel II instrument. cDNA synthesis was performed, as described above. Each 50 μL reaction was composed of 10 μL 5× Induro Buffer (for Induro and LINE-1 RTs), 1 μL 10 μM primer (DNA1_F or IRES_Cas_F; Supplemental Table S1), 1 μg circular RNA template, and nuclease-free water (Invitrogen). Primer and template were annealed at 65°C for 5 min followed by incubation on ice for 5 min. One hundred and twenty-five nanograms of LINE-1/FBu RT, or 1 U of Induro, were added to the reaction, followed by incubation at 37°C for 5 min and incubation at 4°C for 10 min. The reaction was initiated by the addition of 1 mM dNTPs and incubation at 37°C for 3–5 min. Reactions were quenched by transfer to a 4°C metal rack followed by the addition of 5 μL of 10× RT quenching buffer (50 mM Tris pH 8.0, 200 mM NaCl, 50 mM EDTA, 1 μL thermolabile proteinase K [NEB; P8111S]). The solution was incubated at 37°C for 15 min and 55°C for 20 min. Samples were transferred to a 4°C metal rack before dilution of 1:10 in 1× TE buffer and the addition of 1 μL of RNase A (NEB). Samples were incubated at 37°C for 15 min, then cooled to 4°C, pooled in a 1.5 mL tube, and precipitated with ethanol. Pellets were resuspended in 0.0625 volumes of nuclease-free water (Invitrogen) and concentrations were measured using a Qubit ssDNA Assay Kit (ThermoFisher Scientific). Samples were then visualized on a 1% agarose E-gel (ThermoFisher Scientific) for size verification (4–8 kb).

3′ polyadenylation was performed by mixing 5 μL 10× Terminal Deoxynucleotidyl Transferase (TdT) Buffer (NEB), 5 μL 2.5 mM CoCl_2_ (NEB), 5 μL TdT (NEB), and amounts corresponding to 1:1000 cDNA 3′ end:dATP ratio of 1 mM dATP (NEB), and 1 pmol of cDNA. Reactions were incubated for 50 min at 37°C and purified using 1× NEBNext Sample Purification Beads (NEB) and eluted in 34.5 μL nuclease-free water (Invitrogen; AM9932) at 55°C. Second-strand DNA synthesis was performed using Long Amp *Taq* polymerase (NEB) and (8T)VN primer. Reactions were incubated at 65°C for 5 min and cooled down to 4°C on a thermocycler to enable (8T)VN primer annealing [(8T)VN Tm is ∼15°C]. Two microliters of Long Amp *Taq* polymerase (NEB) was added to the reaction before incubation at 65°C for 30 min. DNA end clean up and removal of ssDNA was performed by addition of 1 μL Exo VII exonuclease (NEB) to the reaction mix and incubation at 37°C for 30 min. Samples were purified using 1× NEBNext Sample Purification Beads (NEB) and eluted in 25 μL nuclease-free water (Invitrogen, AM9932) at 55°C. Samples were then analyzed on a TapeStation (Agilent Technologies) using the Genomic DNA ScreenTape kit (Agilent Technologies).

Libraries were prepared from the resulting double-stranded DNA products according to Pacific Biosciences library preparation protocol using the SMRTbell prep kit 3.0 (Pacific Biosciences). After completion of SMRTbell prep, samples were analyzed on a TapeStation (Agilent Technologies) with the Genomic DNA ScreenTape kit (Agilent Technologies). Individual reaction replicates were pooled together in equal molarity, cleaned up in 1.3× SMRTbell cleanup beads, and eluted in 31 μL elution buffer.

To optimize sequencing efficiency and maximize data collection, adapter-ligated DNA was size selected to remove shorter products using 0.75% Agarose Gel Cassette Low Range (Sage Science) on Blue Pippin instrument (Sage Science). The Blue Pippin protocol was run in high-pass mode with a threshold of 3.6 kb minimum. A cleanup using 1.3× SMRTbell cleanup beads was performed, and samples were resuspended in 15 μL of Pacific Biosciences SMRTbell prep kit 3.0 elution buffer and analyzed on a TapeStation (Agilent Technologies) using the Genomic DNA ScreenTape kit (Agilent Technologies). The eluted and purified samples were prepared with the Pacific Biosciences Sequel II binding kit 3.2 (Pacific Biosciences) protocol and loaded at 75 pmol on an SMRT cell 8M Tray (Pacific Biosciences) set to “HiFi Reads.”

#### Second-strand DNA synthesis

Single-stranded cDNA was generated by Induro RT using template DNA1 and purified using the Monarch RNA Purification Kit. One microgram of cDNA was mixed with 1 ng of primer DNA1_R and annealed at 65°C. Annealed DNA was mixed with 1×Induro Buffer followed by the addition of RT enzyme. Samples were incubated at 37°C for 5 min followed by incubation on ice for 10 min. dNTPs were added to 1 mM each and reverse transcription reactions were carried out at 37°C for 30 min. Reactions were purified using the Monarch RNA Cleanup Kit. Pacific Biosciences adapters were then added using the SMRTbell prep kit 3.0 (Pacific Biosciences). Polymerase binding was performed with Pacific Biosciences Sequel II binding kit 3.1 protocol. Samples were loaded onto the Sequel II at a concentration of 75 pmol and with “HiFi Reads” application on an SMRT cell 8M, Tray (Pacific Biosciences).

### Roll-Seq analysis

Following PacBio sequencing, the consensus sequences were built for each ZMW (zero-mode waveguide) output using the PacBio ccs utility. These consensus sequences correspond to long concatemeric reads produced by rolling-circle reverse transcription. Individual concatemers were located by mapping the long concatemeric consensus sequences to the template sequence using the PacBio pbmm2 command-line utility. Based on the mapping coordinates, individual concatemers were extracted, remapped to the template sequence, and all matches, substitutions, deletions, and insertions were called. A few filtering criteria were applied to ensure accurate mutational calls. Each consensus read was required to have at least 15 passes, an overall read quality of 1.0, and the highest mapping quality. The individual concatemers were required to fully map to the template sequence. Only concatemeric reads containing at least three individual concatemers were used for the subsequent downstream analysis. This was done to be able to differentiate between RNA polymerase and reverse transcriptase errors. If a certain error (substitution, deletion, or insertion) was introduced by RNA polymerase, it would be present in every concatemer. On the contrary, if a certain error was introduced by reverse transcriptase, it would be present only once in one of the concatemers. More than one error could be introduced by reverse transcriptase, but they must be present at different positions.

### Second-strand synthesis analysis

Strand-specific consensus sequences were built for each ZMW output from PacBio sequencing. This was achieved by mapping raw sequencing subreads to the reference sequence and splitting subreads corresponding to the opposite strands to separate files. Then the PacBio ccs command-line utility was used to build consensus sequences. Opposite strand consensus sequences were filtered to have at least 15 passes, overall read quality of 1.0, read length within 10 bp of the expected reference sequence length, and expected sequences at both 5′ and 3′ ends. For each ZMW, the cDNA strand was aligned to the second-strand DNA, and the total number of matches, substitutions, deletions, and insertions was calculated.

## DATA DEPOSITION

Roll-Seq sequencing data pertaining to this study have been deposited in the Sequencing Read Archive under accession number PRJNA1101985. Custom software tools are available in the GitHub repository at https://github.com/potapovneb/roll-seq. All requests for materials and data should be addressed to the corresponding authors, J.L.O. or R.J.T.

## SUPPLEMENTAL MATERIAL

Supplemental material is available for this article.

## COMPETING INTEREST STATEMENT

This study was privately funded by New England Biolabs, Inc. Authors V.P., I.U., J.A.B., S.M., S.G., J.L.O., and R.J.T are employees of New England Biolabs, Inc. New England Biolabs is a manufacturer and vendor of molecular biology reagents, including the RNA polymerases and reverse transcriptases used in this study.

## Figures and Tables

**Stanislas Krudup:**
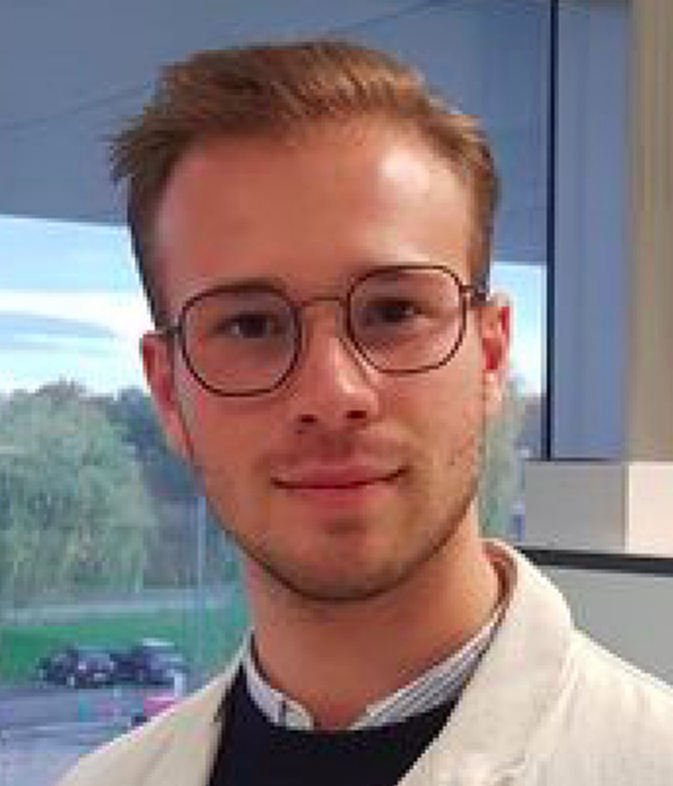


## MEET THE FIRST AUTHORS

***Meet the First Author(s)* is an editorial feature within *RNA*, in which the first author(s) of research-based papers in each issue have the opportunity to introduce themselves and their work to readers of *RNA* and the RNA research community. Vladimir Potapov and Stanislas Krudup are co-first authors of this paper, “Discrete measurements of RNA polymerase and reverse transcriptase fidelity reveal evolutionary tuning.” Stanislas is an engineer in biotechnology and a master in synthetic biology and pharmaceutical management. Stanislas did research on RNA polymerase and reverse transcriptase fidelity assessment before enrolling in brand management and medical affairs in the pharmaceutical and biotech industries.**

**What are the major results described in your paper and how do they impact this branch of the field?**

The development of this new method now allows the evaluation of RNA polymerase and reverse transcriptase mutational activities. It can essentially be used to study other polymerases and their impact on gene mutations.

**What led you to study RNA or this aspect of RNA science?**

**SK:** RNAs are at the heart of metabolic activities and key molecules that are promising for the development of future therapies that will hold the future of the pharma industry, increasing my interest to know more about them.

**During the course of these experiments, were there any surprising results or particular difficulties that altered your thinking and subsequent focus?**

**SK:** The optimization of the protocol and particularly getting the right length of the first single strand of DNA was the most difficult part of the experiments. Indeed, its length depends on the incubation time and was hard to monitor in the beginning.

**If you were able to give one piece of advice to your younger self, what would that be?**

**SK:** Stay curious about everything because a great idea can come from anywhere and from anyone: Creativity is the key but is mastered through rigor.

**What are your subsequent near- or long-term career plans?**

**SK:** I plan on working in the biotech or the pharma industry to take part in the development of promising therapies, even though I am not sure whether it will be as a research manager, at the clinical stages, or at the commercialization stage. However, I have to say that the research stage is by far the most interesting.

**What were the strongest aspects of your collaboration as co-first authors?**

We mostly worked in synergy as I was in the lab and Vladimir was treating the downstream results generated through next-generation sequencing. We then collaborated on the analysis of the results to better understand the phenomenon observed. We also strongly interacted with Dr. Robert Trachman, who successfully led the project.
